# Methyl-donor supplementation prevents intestinal colonization by Adherent-Invasive *E. coli* in a mouse model of Crohn’s disease

**DOI:** 10.1038/s41598-020-69472-3

**Published:** 2020-07-31

**Authors:** Elodie Gimier, Mélissa Chervy, Allison Agus, Adeline Sivignon, Elisabeth Billard, Maud Privat, Sandrine Viala, Régine Minet-Quinard, Anthony Buisson, Emilie Vazeille, Nicolas Barnich, Jérémy Denizot

**Affiliations:** 1 Université Clermont Auvergne, Inserm U1071; USC-INRAE 2018, Microbes, Intestin, Inflammation et Susceptibilité de l’Hôte (M2iSH), 28 Place Henri Dunant, 63001 Clermont-Ferrand, France; 2 U1240 Imagerie Moléculaire et Stratégies Théranostiques, Université Clermont Auvergne, INSERM, 63000 Clermont-Ferrand, France; 3 Département d’Oncogénétique, Centre Jean Perrin, 63000 Clermont-Ferrand, France; 4 Service de Biochimie et Génétique Moléculaire, CHU Clermont-Ferrand, 63000 Clermont-Ferrand, France; 5 CNRS UMR 6293, INSERM U1103, GReD, Université Clermont Auvergne, 63000 Clermont-Ferrand, France; 6 Service d’Hépato-Gastro Entérologie, 3iHP, CHU Clermont-Ferrand, 63000 Clermont-Ferrand, France; 7 Present Address: Micalis Institute, Institut National de la Recherche Agronomique (INRAE), AgroParisTech, Université Paris-Saclay, 78352 Jouy-en-Josas, France

**Keywords:** Crohn's disease, Cellular microbiology, Preclinical research, Bacterial infection

## Abstract

Deficiencies in methyl-donor molecules (folate, B12 vitamin), DNA methylation alteration and high prevalence of Adherent-Invasive *Escherichia coli* (AIEC) are frequently observed in Crohn’s disease (CD) patients. AIEC bacteria adhere to the enterocytes through abnormally expressed carcinoembryonic antigen-related cell adhesion molecule 6 (CEACAM6) glycoprotein on host cells. This work aims at studying the relationship between methyl-donor molecules and AIEC-induced intestinal inflammatory response. CEABAC10 mice, a mouse model of CD, were fed a control or Methyl-donor Supplemented diet (MS diet). *CEACAM6* promoter was hypermethylated in intestinal epithelial cells from mice fed an MS diet, which was associated with a significant decrease in *CEACAM6* expression. Transcriptomic analysis revealed increased expression of anti-microbial peptides, increase in HSP70 gene family expression and a decreased expression of inflammatory marker Calprotectin upon MS diet, associated to a lower ability of AIEC bacteria to colonize gut mucosa. We observed in a cohort of CD patients that serum folate concentration was inversely correlated to Crohn’s disease endoscopic index of severity and to fecal inflammatory markers. This study demonstrates that methyl-donor supplementation through the diet induces a specific intestinal micro-environment limiting pathobiont colonization of the gut. Clinicians may wish to consider methyl-donor supplementation for methyl-donor deficient CD patients.

## Introduction

Crohn’s Disease (CD), an Inflammatory Bowel Disease (IBD), is a chronic inflammatory disorder of the gastrointestinal tract, affecting 2 million people in Europe with a rising incidence in newly industrialized countries^1^. Its etiology involves environmental factors, like the western lifestyle, genetic and microbial factors, leading to abnormal immune response of intestinal mucosa^2,3^. Recently, studies have highlighted alteration of DNA methylation in CD patients, which could be one of the missing links between intestinal inflammatory phenotype in CD patients and environmental factors. During the last decade, specific attention was given to the study of DNA methylation in IBD patients’ blood cells and intestinal epithelial cells, all showing differentially methylated CpG sites in CD patients, when compared to controls^4–13^. DNA methylation occurs within the one-carbon metabolism pathway and is dependent on several enzymes (dihydrofolate reductase, methylene-tetrahydrofolate reductase, methionine synthase) and on the availability of micronutrients used as cofactors such as folate (B9 vitamin), B12 vitamin (methionine synthase cofactor), methionine, choline and betaine. Folate enters the one-carbon metabolism cycle where it is converted, as a result of many enzymatic reactions, into S-adenosyl-methionine used as a methyl-donor by DNA Methyltransferase (DNMT) enzymes to catalyze the DNA methylation reaction^14^. The level of folate and other methyl-donor molecules intake through the diet were associated to the regulation of DNA methylation. Even more significantly, serum B12 and folate (B9) deficiencies have frequently been noticed in different cohorts of IBD patients^14–20^. Based on the known roles of these molecules in one-carbon metabolism, epigenetic modifications such as DNA methylation profiles and modified genes expression observed in CD patients could be the result, in part, of methyl-donor molecules deficiencies.

A high prevalence of invasive *Escherichia coli* strains (21–63% of CD patients), designated as the pathotype Adherent-Invasive *E. coli* (AIEC), has been detected in the ileal mucosa of CD patients in many studies worldwide^21–26^. These bacteria induce secretion of pro-inflammatory cytokines and intestinal inflammation in a genetically susceptible mouse model^27^. AIEC can adhere to and invade intestinal epithelial cells (IECs), through the interaction with the abnormally over-expressed mannosylated CEACAM6 protein in IECs of CD patients^28,29^. We used the CEABAC10 mouse model to study AIEC intestinal colonization in vivo. This transgenic mouse model carries 4 human CEACAMs genes (*CEACAM3, CEACAM5, CEACAM6* and *CEACAM7*) under the control of their human promoter. As previously described by our group, AIEC bacteria highly colonize the gut mucosa of these mice in comparison to WT mice, showing that this mouse model is appropriate to mimic AIEC colonization in CD^27,30^. We previously highlighted a DNA methylation-dependent regulation of *CEACAM6* gene transcription. Methylation of a specific CpG within the *CEACAM6* gene promoter impairs HIF-1 transcription factor binding, controlling the transcription of the gene^31^. As CD patients frequently present defects in methyl-donor molecules and in DNA methylation pattern, one strategy to limit AIEC colonization could be to restore the methylation pattern of *CEACAM6* (and other misregulated genes) to decrease its expression through diet-based strategy by increasing intake in methyl-donor molecules. Our hypothesis is that methyl-donor supplementation, such as folate and B12 vitamin could modulate gene expression in IECs, decrease *CEACAM6* gene expression and, therefore, prevent AIEC colonization and subsequent inflammation.

Methyl-donor enriched or deficient diets have been used in many research contexts. These studies have genuinely demonstrated that maternal-methyl-donor supplementation increases DNA methylation in the offspring and sensitivity of mice to DSS-induced colitis^32–35^. In contrast, methyl-donor deficiency leads to a decrease in DNA methylation associated to a weaker intestinal barrier function and also leads to an increase in the sensitivity of rats to DSS-induced colitis, suggesting a central role of methyl-donor molecules during the course of inflammation^36–38^. However, no studies have analyzed the effect of a methyl-supplemented diet during CD-associated pathobiont bacterial challenge. Our hypothesis is that a methyl-donor supplementation through the diet could limit AIEC bacteria intestinal colonization in the well-established CEABAC10 mouse model of CD through the modulation of DNA methylation. This study establishes a relationship between methyl-donor molecules and intestinal inflammation in the context of AIEC colonization and in a cohort of CD patients.

## Results

### Addition of methyl-donor molecules in the diet decreases CEACAM6 gene expression

**Figure 1:**
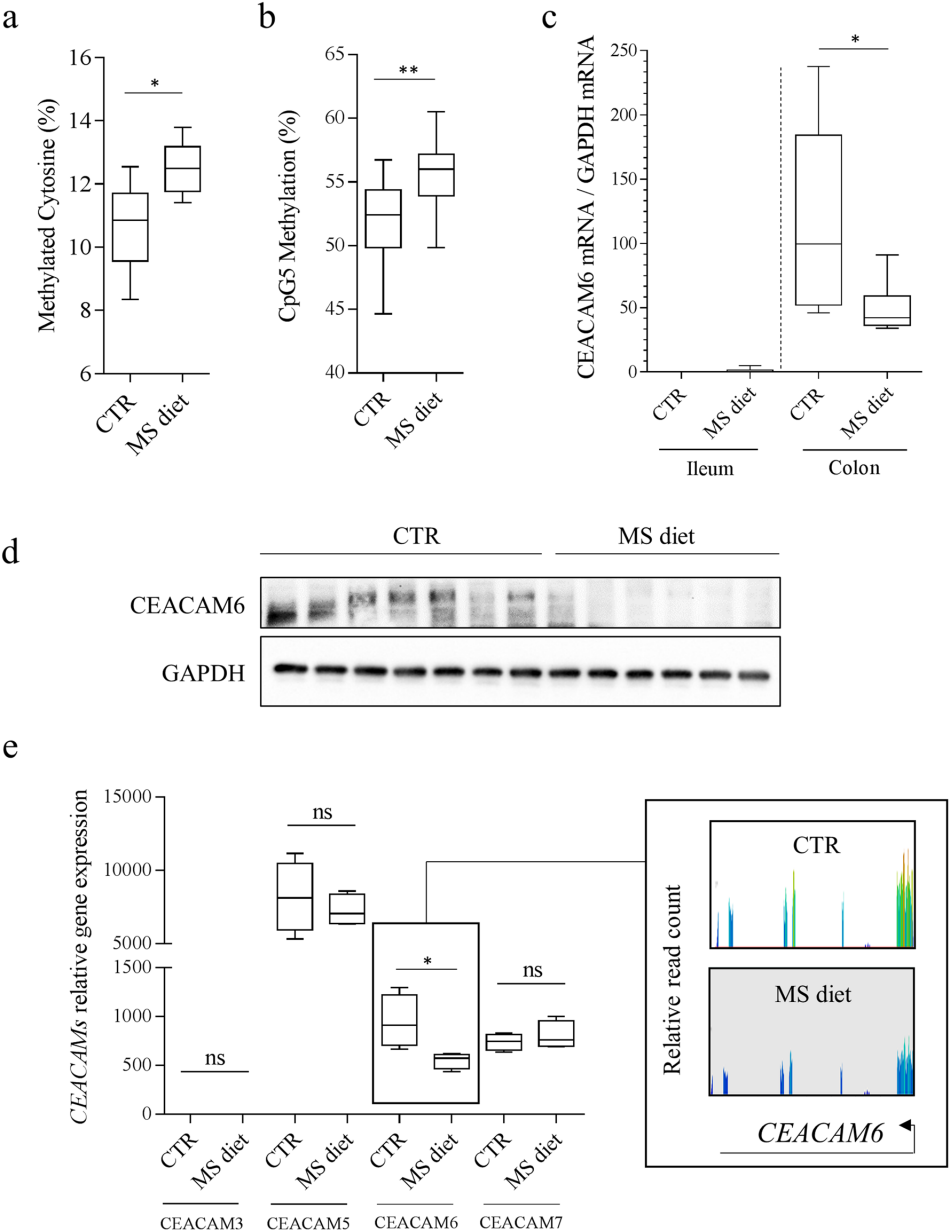
Methyl-donor supplementation decreases *CEACAM6* expression through hypermethylation of its promoter in intestinal epithelial cells in vivo. (**a**) Cytosine methylation levels on LINE elements from colonic mucosa of mice fed a control (CTR) or Methyl-Supplemented diet (MS diet). (**b**) Methylation level of CpG5 (within HIF-1 responsive element) within *CEACAM6* promoter was measured on purified enterocytes from mice fed a control diet (CTR) (n = 12) and MS diet (n = 10) using Bisulfite-SnapShot. (**c**) *CEACAM6* mRNA was quantified by RT-qPCR in ileal and colonic mucosa (n = 4). (**d**) Western blot performed on colonic mucosa of CEABAC10 mice fed a CTR or MS diet for quantification of CEACAM6 expression (n = 7 and 6 respectively). The two signals were obtained from the same gel migration. The membrane was cropped as the anti-CEACAM6 antibody was made in mice; hence the secondary antibody recognizes the heavy and light chains of endogenous IgG, masking the CEACAM6 signal. Uncropped membranes are shown in Supplementary Figure S1. (**e**) *CEACAMs* genes expression encoded by the CEABAC transgene was measured in colonic mucosa from mice fed a control diet (CTR) (n = 4 biological replicates) and MS diet (n = 4 biological replicates) by RNA-seq. Insert: Screen-shot of quantified CEACAM6 reads from RNA-seq data. Mann–Whitney test. *ns* non-significant. *p < 0.05; **p < 0.01.

Adherent-Invasive *E. coli* are frequently found in ileal lesions in CD patients and use CEACAM6 as a receptor for their adhesion and entry within IECs. *CEACAM6* was previously identified as a gene regulated by DNA methylation on a specific CpG site (named CpG5) within a Hypoxia Inductible Factor (HIF)-1 Responsive Element^31^. Mice were fed a diet enriched in methyl-donor molecules [Methyl-donor Supplemented diet (MS diet)] to increase global DNA methylation in intestinal epithelial cells, as previously described^32,33^. As expected, we observed a significant increase in the proportion of methylated cytosine on LINE (Long Interspersed Nuclear Element) in colonic mucosa from mice fed an MS diet, compared to mice fed the control diet (CTR diet) (CTR: 10.68%; MS diet: 12.48% *p* = 0.0488, n = 5) (Fig. 1a). Then, we specifically assessed the effect of MS diet on *CEACAM6* promoter methylation and gene expression in intestinal mucosa of mice receiving a control diet or an MS diet by Bisulfite-SnapShot, RT-qPCR, western blot and RNA-seq. We observed an increase in CpG5 methylation level (which is found in a binding site for HIF-1 transcription factor^31^) in colonic mucosa of mice fed an MS diet when compared to mice fed a control diet (*p* = 0.010) (Fig. 1b). RT-qPCR analysis showed a significant decrease in *CEACAM6* mRNA level in colonic mucosa of CEABAC10 mice fed an MS diet, compared to mice fed a control diet (mean CTR: 114.4, n = 5; mean MS diet: 49.15, n = 6 ANOVA, *p* < 0.05) (Fig. 1c). This observation was confirmed by western blot (Fig. 1d and Supplementary Figure S1). Interestingly, RNA-seq analysis revealed no change in the expression levels of three of the genes encoded by the transgene (humans *CEACAM3*, *CEACAM5* and *CEACAM7*) in intestinal mucosa from CEABAC10 mice fed an MS diet. A significant 1.72-fold decrease in *CEACAM6* expression in intestinal mucosa of mice fed an MS diet was observed, compared to mice fed the conventional diet (*p* = 0.029) (Fig. 1e). Taken together, these observations reveal that methyl-donor molecules supplementation in the diet could decrease *CEACAM6* gene expression in vivo, which could be of interest in the treatment of Enterobacteria-colonized CD patients.

### RNA-seq analysis indicates a protective gene expression profile upon MS diet feeding in colonic mucosa

**Figure 2:**
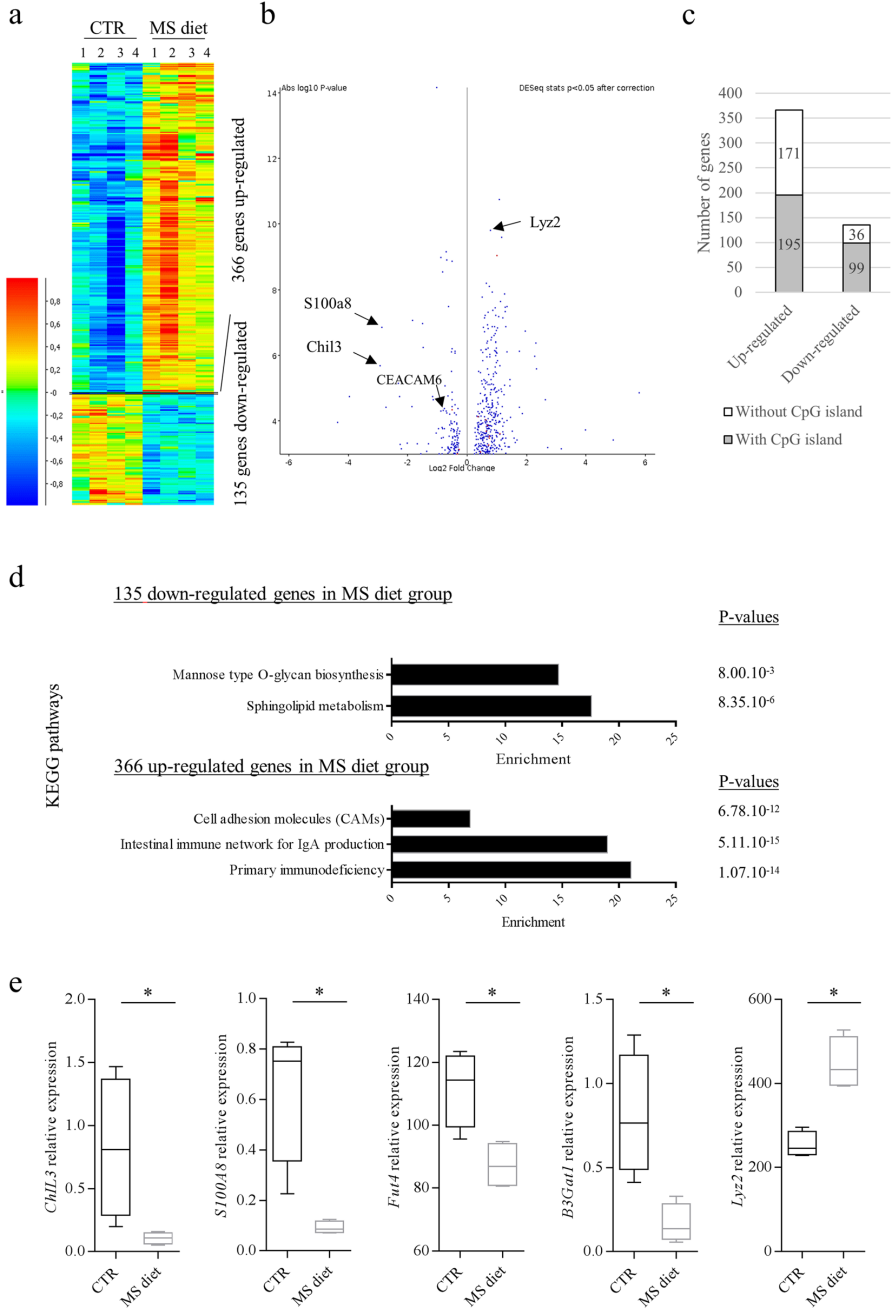
Transcriptomic profile of colonic mucosa upon MS diet feeding. mRNA-seq was performed on RNA from colonic mucosa (n = 4 for CTR, n = 4 for MS diet). (**a**) Heat-map showing the differentially expressed genes between the both groups (DeSeq2 stat, *p* < 0.05). (**b**) Volcano plot showing the significantly misregulated genes in MS diet, compared to control diet. (**c**) Number of misregulated genes harboring CpG islands within their promoter. (**d**) KEGG pathways analysis of up- and down-regulated genes in MS diet group, compared to control diet-fed group. (**e**) Quantification of expression levels of 5 genes of interest: *chitinase like 3*, *S100A8* (Calprotectin subunit), *Fut4, B3gat1* and *Lyz2* genes. Mann–Whitney test. *p < 0.05.

To assess global changes in gene expression in response to MS diet, we performed RNA-seq analysis on colonic mucosa from mice fed a control diet and mice fed an MS diet. DeSeq2 statistical analysis identified 135 genes as significantly down-regulated and 366 genes as significantly up-regulated in mice fed an MS diet compared to mice fed a control diet (*p* < 0.05, n = 4) (Fig. 2a, b). Interestingly, among the 135 down-regulated genes, 73.3% (99 genes) are known as regulated by a CpG island in their promoter whereas among the 366 up-regulated, 53.3% of them are regulated by a CpG island in their promoter (Fig. 2c). These data suggest that MS diet preferentially leads to a decrease in expression of genes harboring a CpG island within their promoter, as expected. Among the 135 down-regulated genes, gene ontology analysis associated these genes to two pathways as sphingolipid metabolism and mannose-O-glycan biosynthesis (*Fut4, B3Gat1*), which could lead to modifications of glycosylation of intestinal mucosal surface (Fig. 2d, e and Supplementary Tables S1 and S3). A down-regulation of Calprotectin subunits S100a8 and S100a9 (markers of inflammation) appeared in mice fed an MS diet though, which confirms an obvious relationship between methyl-donor molecules and the regulation of pro-inflammatory markers expression (Fig. 2b, e). In contrast, among the 366 up-regulated genes, antibacterial genes such as Lyz1 and Lyz2 were identified, suggesting a potential better ability of intestinal mucosa to counteract bacterial infection (Fig. 2b, e and Supplementary Tables S2 and S3). Interestingly, we observed an increased expression of heat-shock protein genes HSPA1A and HSPA1B, members of HSP70 family, upon MS diet feeding (Supplementary Table S2). These genes have clearly been shown as protective against intestinal inflammation in different models (see discussion section). It is worth to note that 15 of the up-regulated genes were associated to “Intestinal immune network for Secretory IgA (SIgA) production” (*p* value = 5.11 × 10^–15^, enrichment = 20.2) (Fig. 2d). RNA-seq revealed a 1.55 fold increase in the J chain gene expression (*p* = 9.52 × 10^–5^), which contributes to the formation and secretion of Secretory Immunoglobulin A (SIgA), in mice fed an MS diet. A higher secretion of SIgA within colonic lumen of MS diet-fed mice could therefore modify intestinal microbiota composition and could favor elimination of pathogenic/pathobiont bacteria, such as AIEC in CD patients.

### MS diet impacts IgA concentration in the lumen

**Figure 3:**
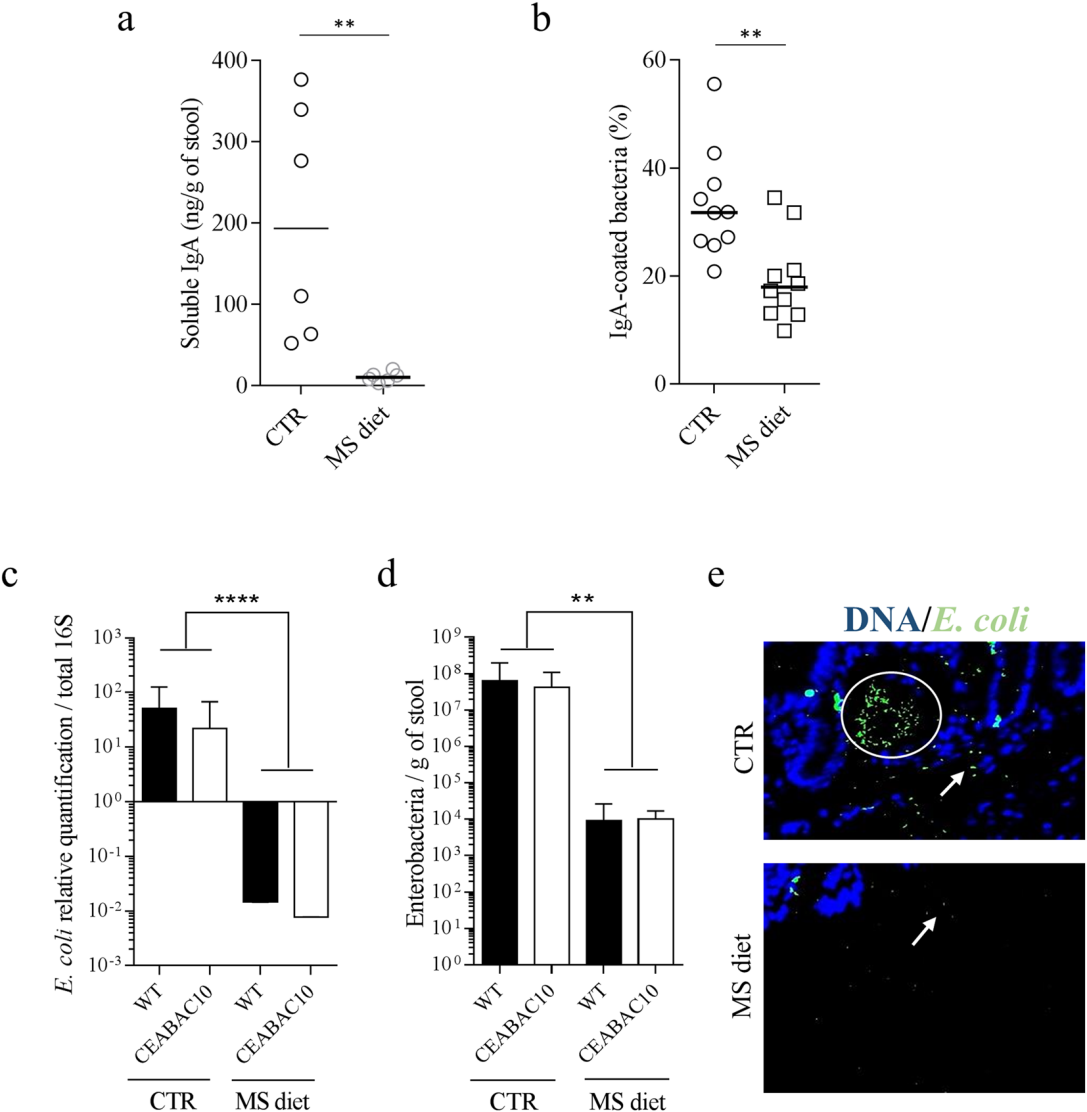
MS diet decreases SIgA secretion and *E. coli* load in mice stools. (**a**) ELISA quantification of SIgA in fecal pellet of mice fed a control or MS diet (n = 6). (**b**) SIgA-coated bacteria were quantified in the stools of mice using flow cytometry. Anti-SIgA-PE antibody was used to detect IgA + bacteria. The data represent the percentage of IgA + bacteria in control diet (CTR) and MS diet groups, over the total number of events detected (n = 10 for each group). (**c**) qPCR relative quantification of *E. coli* in stools, standardized to the total copy number of 16S in mice fed CTR (n = 16) and MS diet (n = 16). (**d**) Quantification of Gram negative bacilli count on Drigalsky agar plate in stools from mice fed CTR (n = 16) and MS diet (n = 16). (**e**) Immunofluorescence staining of *E. coli* associated to colonic mucosa. *E. coli* bacteria were stained in green, DNA was stained in blue. White arrows indicate *E. coli* bacteria. Mann–Whitney test. *ns* non-significant. **p < 0.01; ****p < 0.0001.

To analyze the relevance of RNA-seq data, we quantified the SIgA concentration in the stools of mice fed a control diet and an MS diet. In contrast to what we expected, based on RNA-seq data, we observed a huge decrease in SIgA quantity in the stools of CEABAC10 mice fed an MS diet (CTR: 203.20 ng/g; MS diet: 10.50 ng/g, *p* = 0.022, n = 6) (Fig. 3a). Then, we analyzed the proportion of SIgA-coated bacteria in the stools of mice fed the control or the MS diet using flow cytometry. SIgA-coated bacteria were stained with an anti-SIgA antibody coupled to PE fluorochrome. We observed a significant decrease in the proportion of SIgA-coated bacteria in mice fed an MS diet compared to control diet (CTR mean: 33.36% vs MS diet 19.49%; *p* = 0.004) (Fig. 3b). These data suggest that MS diet leads to a fewer number of SIgA-coated bacteria in the microbiota, which could be a response to drastic changes in microbiota composition upon MS diet feeding.

### MS diet prevents commensal *E. coli* overgrowth in colonic mucosa

As we observed a decrease in glycosylation pathways (essential for bacterial growth and attachment), and a decrease in SIgA secretion upon MS diet feeding, we analyzed the fecal *E. coli* load in mice fed a control diet or an MS diet. qPCR approach revealed a 3.3 × 10^3^-fold decrease in *E. coli* population in mice fed an MS diet when compared to mice fed a control diet (*p* < 0.001) (Fig. 3c). These molecular data were confirmed by cultural approach by quantification of Enterobacteria on Drigalsky agar plate (selective for Gram negative bacteria) where we observed a 5.8 × 10^3^-fold decrease in Enterobacteria load in mice fed an MS diet (*p* = 0.003), independently of the genotype of the mice (Fig. 3d). Immunostaining on colonic section confirmed these observations. Very few *E. coli* were observed associated to colonic mucosa or in the lumen of MS diet-fed mice whereas *E. coli* population was abundant in control diet-fed mice (Fig. 3e).

### MS diet protects against pathobiont AIEC infection in vivo

**Figure 4:**
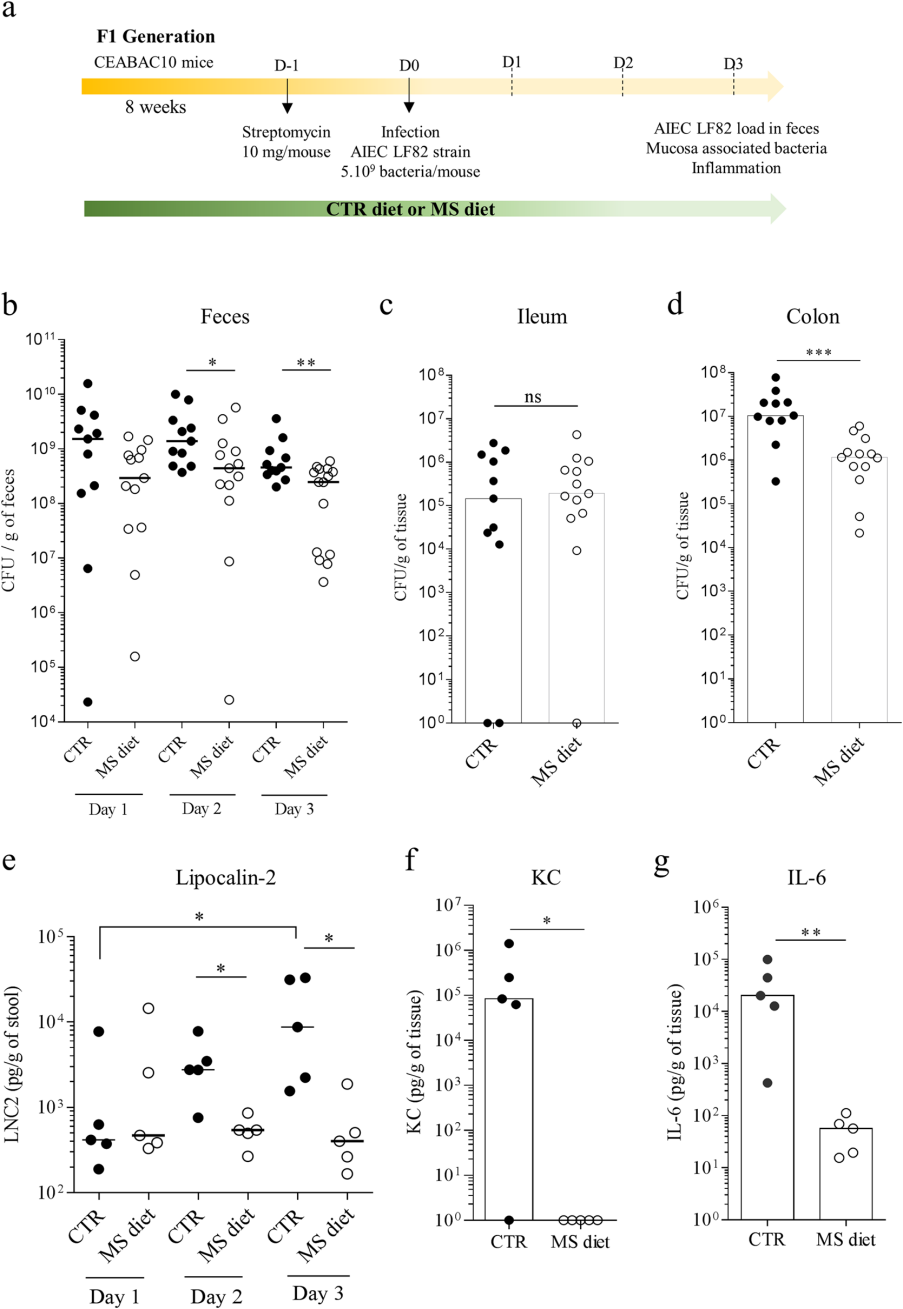
MS diet protects mice from AIEC intestinal colonization and inflammation. (**a**) Experimental protocol of infection used in the study. (**b**–**d**) AIEC LF82 load in (**b**) the stools at days 1, 2 and 3 post-infection, (**c**) associated to ileum and (**d**) associated to colonic mucosa was quantified on selective medium (CTR group: n = 11, MS diet: n = 13–15, two independent experiments pooled). (**e**) Micro-inflammation marker Lcn-2 was quantified in the stools, at days 1, 2 and 3 post-infection by ELISA (CTR group: n = 5, MS diet: n = 5). (**f**–**g)** Pro-inflammatory cytokines KC (Keratinocyte Chemoattractant) (**f**) and IL-6 (**g**) were quantified in colonic mucosa release medium by ELISA (n = 5 for each group). Mann–Whitney test. *p < 0.05; **p < 0.01; ***p < 0.001.

As we observed that MS diet decreases commensal *E. coli* bacteria in the gut microbiota, and decreases AIEC receptor *CEACAM6* gene expression, we hypothesized that MS diet could prevent pathobiont AIEC bacteria encroachment and subsequent inflammation by inducing a specific intestinal micro-environment. To test this hypothesis, control or MS diet–fed CEABAC10 mice were orally challenged by AIEC reference strain LF82 (Fig. 4a). AIEC LF82 count in the stools did not reveal any differences between the both groups at day 1 post-infection. In contrast, we observed a significant 2.55-fold (*p* = 0.031) and a 2.57-fold (*p* = 0.009) decrease in AIEC load in MS diet-fed mice compared to control diet-fed ones 2 and 3 days post infection, respectively (Fig. 4b). Nevertheless, no difference was observed in the number of ileum-associated AIEC LF82 between the groups (Fig. 4c). These data agree with the due attention that *CEACAM6* gene is not expressed in ileal mucosa in this model (Fig. 1c). Nonetheless, a 11.51-fold decrease was noted in the number of bacteria associated to colonic mucosa of mice from the group fed MS diet compared to mice fed a control diet (*p* < 0.001) (Fig. 4d). These data show that the micro-environment established by MS diet is not favorable for AIEC bacteria attachment and long-term colonization.

To better characterize the inflammatory response induced during the course of infection, we quantified the micro-inflammatory marker lipocalin-2 (Lcn-2) in the stools, during infection. We observed a significant increase in Lcn-2 levels, throughout the infection study, in mice fed control diet (from day 1 to day 3 post-infection). In contrast, no changes in Lcn-2 levels were measured in MS diet-fed mice during the course of infection (Fig. 4e). Lcn-2 level was 23.85 times lower in MS diet-fed mice compared to control diet-fed mice at day 3 post infection (*p* = 0.0159). The analysis of pro-inflammatory cytokines release from colonic tissues showed a significant decrease in Keratinocyte Chemoattractant protein (KC) (*p* = 0.048) and in IL-6 secretion (*p* = 0.008) in mice fed an MS diet compared to mice fed a control diet (Fig. 4f–g). These data support the protective role of MS diet in the context of AIEC infection.

### Serum B9 vitamin/Folate concentration is inversely correlated to intestinal inflammation in CD patients

**Figure 5:**
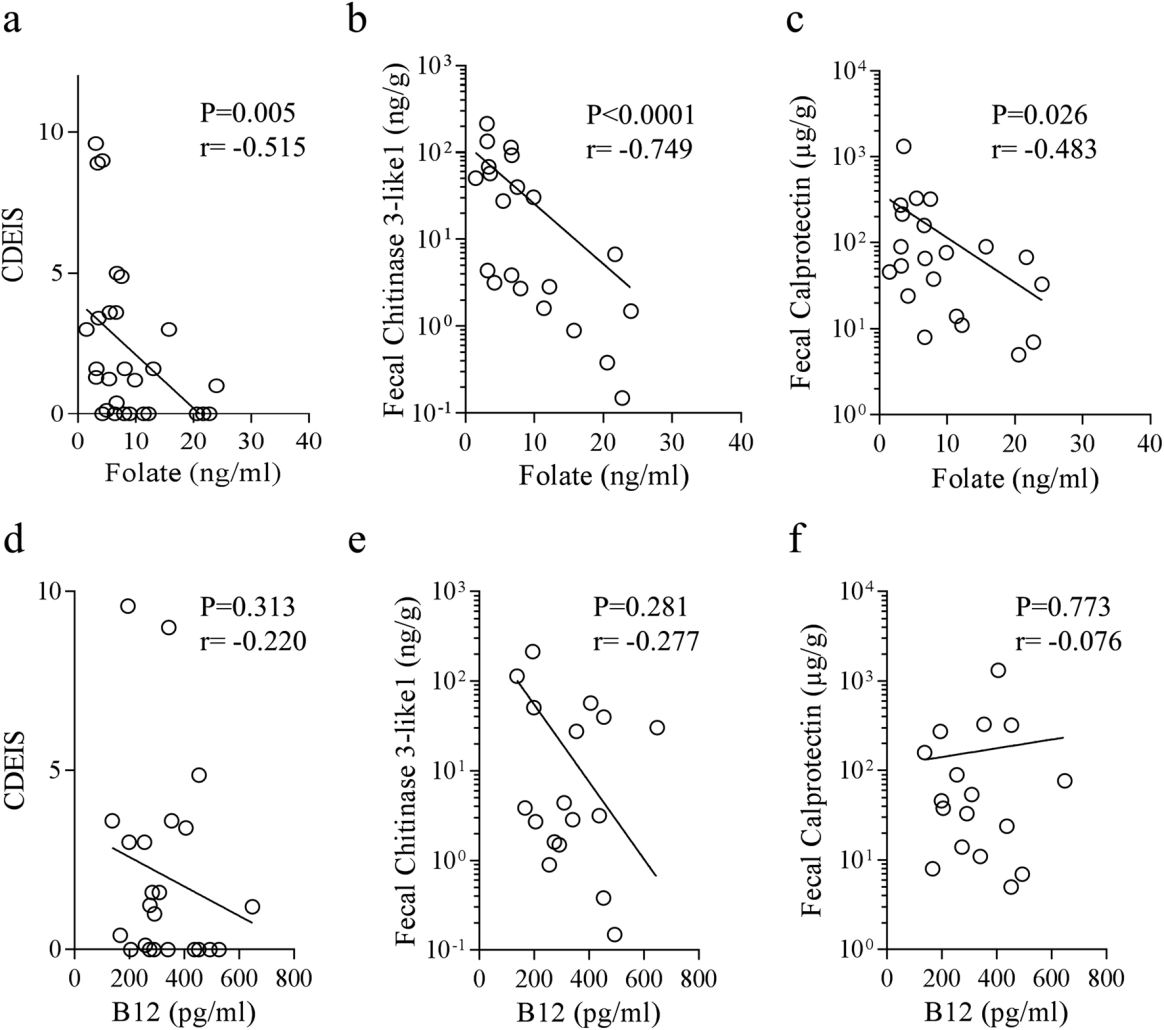
Serum folate level, but not B12 level, is inversely correlated to Crohn’s disease endoscopic index of severity (CDEIS) and to fecal markers of inflammation. B9 (folate) and B12 vitamins were quantified in the sera of CD patients (n = 28 patients). (**a**, **d**) Correlation between CDEIS and serum vitamins level at time of endoscopy in CD patients. (**b**, **e**) Correlation between fecal Chitinase 3-Like 1 level and serum vitamins concentration in CD patients (n = 21 pairs). (**c**, **f**) Correlation between fecal Calprotectin level and serum vitamins concentration in CD patients (n = 21 pairs). Spearman test was used to assess the correlation existing between the two variables tested.

CD patients frequently present deficiencies in B9 (folate) and B12 vitamins. However, the interconnection between serum folate levels and intestinal inflammation in CD patients remains unclear. To answer this question, we used a previously established CD patients’ cohort in the laboratory to quantify folate (B9 vitamin) and B12 vitamin in the serum (Table 1). The levels of folate ranged from 1.49 to 23.99 ng/ml, with a median of 9.01 ng/ml. Six patients out of 28 presented low folate levels (under 4.00 ng/ml), representing 21.43% of patients from our cohort. We determined whether correlation between folate levels and disease parameters exist. No correlation between folate levels and CDAI, disease location nor active smoking was identified in our cohort. Interestingly though, a significant inverse correlation between Crohn’s disease endoscopic index of severity (CDEIS) and folate (n = 28 pairs, Spearman r = − 0.515, *p* = 0.0048) was uncovered (Fig. 5a). This finding suggests that a link could exist between folate level and endoscopic score in CD. To better characterize the relationship between folate and inflammation, Chitinase 3-Like 1 (Chi3-L1) and Calprotectin, two fecal inflammatory markers, were also quantified in the feces from the same patients^39^. It appeared that folate levels were clearly inversely correlated to Chitinase 3-Like 1 concentration in CD patients’ feces (n = 21 pairs, Spearman r = − 0.749; *p* < 0.0001) (Fig. 5b). Folate levels were also inversely correlated to fecal Calprotectin concentration in CD patient’s feces (n = 21 pairs, Spearman r = − 0.483; *p* = 0.026) (Fig. 5c). The patients presenting high levels of folate show the lowest levels of inflammatory markers in feces. In contrast, no significant inverse correlation was found between B12 vitamin concentrations and the inflammatory parameters we analyzed (Fig. 5d–f). These data imply that folate, a major methyl-donor molecule, could be predictive of endoscopic lesions and could play a role in the control of intestinal inflammation in CD patients.

**Table 1:** Baseline characteristics of the 28 patients with CD enrolled in the study.

	Overall population
	n = 28
Age at inclusion (years), mean ± SD	38.07 ± 13.1
Female gender, n (%)	19 (67.9%)
Active smokers, n (%)	7 (25.0%)
**Montreal classification**	
*CD location*	
L1, n (%)	10 (35.7%)
L2, n (%)	3 (10.7%)
L3, n (%)	14 (50.0%)
*CD behaviour*	
B1, n (%)	21 (75.0%)
B2, n (%)	4 (14.3%)
B3, n (%)	2 (7.1%)
**Current medications**	
5-ASA	2 (7.1%)
Corticoids	1 (3.6%)
Thipurines	4 (14.3%)
Methotrexate	1 (3.6%)
Infliximab	2 (7.1%)
Adalimumab	4 (14.3%)
Vedolizumab	0 (0.0%)
CDAI, mean ± SD	105.0 ± 80.95
CDEIS, mean ± SD	2.3 ± 2.9
Calprotectin, mean (µg/g) ± SD	154.81 ± 286.16
Chitinase 3-Like 1, mean (ng/g) ± SD	40.96 ± 56.95

*SD* standard deviation, *n* number, *CD* Crohn’s disease, *CDAI* Crohn Disease Activity Index, *CDEIS* Crohn’s disease endoscopic index of severity, *5-ASA* acide 5-aminosalicylique.

## Discussion

Patients with Crohn’s disease frequently present vitamin deficiencies such as vitamin B9 (Folate) and vitamin B12, as reported in many studies around the world^15–19^ and recently confirmed in a review of meta-analyses by Piovani et al.^40^. Folate deficiencies in patients were associated with greater disease activity in a Spanish cohort^19^. Moreover, metabolomic study of serum samples from pediatric CD patients revealed amino acid metabolism, folate biosynthesis and signaling pathways as profoundly altered^41^.

Folate and other methyl-donor molecules play a crucial role in the maintenance of epigenetic marks such as DNA methylation. The importance of the epigenetic component in the etiology of CD has recently been illustrated by the demonstration of modified DNA methylation profiles in patients compared to healthy subjects^5,7,9,12,42^. Furthermore, polymorphisms in the *DNMT3a* gene (involved in the establishment of de novo DNA methylation marks) were previously associated to an increased risk to develop CD, suggesting an important role of DNA methylation in the inflammatory process^43^. As previously reported, the *CEACAM6* gene that promotes the adhesion of AIEC bacteria to the intestinal epithelium, is a key gene in CD regulated in a methylation-dependent manner^27,31^. These data from the literature led the study moving on the hypothesis that a diet supplemented in methyl-donor molecules (MS diet) could be used to favor methylation of the *CEACAM6* promoter gene (among others). If so, this could lead to the decrease of *CEACAM6* expression in IECs, thereby limiting susceptibility of CD patients to be colonized by pathobiont bacteria, such as AIEC. To determine whether enrichment in methyl-donor molecules in the diet could modulate the expression of the *CEACAM6* gene, mice either received a control diet or an MS diet during the pregnancy. The analysis was carried out on the offspring, as previously reported in many studies interested in the effect of the diet on the establishment of DNA methylation marks^32,35,44,45^. The contribution of methyl-donor molecules group is attested here by the downregulation of *CEACAM6* gene expression in intestinal epithelial cells in vitro and in vivo. Note that in our transgenic mouse model, the human CEACAMs genes are under the control of their own human promoter, allowing the study of their regulation in vivo^30^. The supplementation of methyl-donor molecules by the diet is sufficient to methylate the *CEACAM6* gene promoter and to limit its expression. This was confirmed and demonstrated in vivo by an increase in the methylation of the HIF-1 transcription factor binding site, known to participate in gene transcription activation^31^. It is interesting to note that the expression of the other *CEACAMs* genes on the transgene, and in the genome, did not show any modification of their expression in response to the MS diet, suggesting methylation-independent regulation mechanisms for these genes. However, RNA-seq analysis has highlighted many genes regulated by the MS diet. The KEGG pathway analysis revealed several significantly modified biological pathways in response to the MS diet. As an example, genes involved in the “Mannose-O-type glycan biosynthesis” pathway *B3gat1* and *Fut4* were down-regulated in the MS diet group. Glycosylation mechanisms are indeed important in the context of adhesion of AIEC bacteria to the intestinal epithelium as the bacterial FimH adhesin recognizes mannose residues attached on CEACAM6 protein, thus allowing the adhesion of the bacterium. AIEC also bind to the intestinal epithelium through the interaction between bacterial ChiA and intestinal N-glycosylated Chitinase 3-Like 1 protein^28,46,47^. Hence, decreasing glycosylation at the surface of the intestinal epithelium may be relevant to limit colonization by AIEC bacteria. The study of glycosylation of proteins in response to the MS diet deserves attention in future studies. The RNA-seq analysis also revealed an increase in the synthesis of Lyzozyme 1 and 2, which are antimicrobial peptides^48^, as well as a decrease in the expression of the two inflammatory markers Chi3-L1 and Calprotectin (*S100a8*)^39^. Moreover, we observed an increase in *Hspa1a* and *Hspa1b* gene expression, two members of HSP70 family. Ohkawara et al.^49,50^ have shown that mice expressing high levels of HSP70 and HSP40, could resist to DSS-induced colitis. In contrast, inhibition of HSPs led to the opposite phenotype and resulted in the development of colitis. These observations suggest that methyl-donor molecules instigate a potential anti-bacterial transcriptomic program in colonic mucosa.

We have demonstrated here that the specific intestinal context induced by MS diet limited intestinal colonization by AIEC bacteria and controlled the inflammation induced during the course of the infection. One explanation could be that MS diet induces many changes in gene expression in intestinal mucosa, rendering the host more prone to counteract specific infectious agents. We observed an increase in the expression of genes involved in the synthesis pathway of SIgA. SIgA are dimeric immunoglobulins secreted in the mucus and known to limit the accession of bacteria to the intestinal epithelium^51,52^. It has been observed in murine models that SIgA preferentially target bacteria with colitogenic potential^53^. Despite the increased expression of these genes, fewer bacteria were coated with these antibodies in MS diet group, suggesting a less colitogenic microbiota in MS diet group compared to control group. Viladomiu et al.^54^ observed a selective enrichment in IgA-coated Adherent-Invasive *E. coli* in patients with CD-associated spondylarthritis compared to CD alone without spondylarthritis. This observation suggests that AIEC bacteria can be targeted by SIgA in an inflammatory context and that stimulating this pathway could lead to a decrease AIEC load in CD patients. The levels of soluble IgA and the percentage of IgA-coated bacteria strikingly increases in feces of IBD patients and correlates with the disease activity^55–57^. This could be related to an over-activation of IgA-producing mucosal B-cells in response to bacterial stimulus of the adaptive immune system. In our study, the MS diet limited the proportion of bacteria coated with IgA, which was associated with a sharp decrease in *E. coli* population. It is likely that the micro-environment induced by the diet is unfavorable to the development of *E. coli* bacteria or favorable to their elimination and that such an environment could benefit to CD patients highly colonized by AIEC bacteria.

Finally, all the experiments presented here did not identify the methyl-donor molecule involved in the protective phenotype observed as this was not the main objective of this study. It is not clear whether the pool of methyl-donor molecules used is necessary to reach a protection against AIEC colonization or whether only one molecule is sufficient. This will be tested in a future study. Based on the literature, one of the most promising molecules is the zinc. Zinc deficiency is frequently observed in IBD patients with a prevalence ranging from 15 to 40%^58–60^. Pre-clinical studies highlighted that decreased zinc concentration in the serum is associated to increased inflammation through alteration of epithelial barrier function and increased pro-inflammatory cytokines IL1-β and IL-6 in response to LPS stimulation, through promoter demethylation (for review^60^ and^61^). However, it is likely that other molecules from the MS diet (betaine, folate, B12 vitamin, biotin, and methionine) all play a role in the regulation of genes expression and protection against AIEC through different but complementary mechanisms. Interestingly, in our study, we revealed for the first time, a statistically significant inverse correlation between serum folate and CDEIS. Besides, serum folate levels were inversely correlated with the concentration of inflammatory markers in the feces (Chitinase 3-Like 1 and Calprotectin). These data point out that the level of serum folate could be used, with the markers already described, as a biomarker of the disease activity.

**Figure 6:**
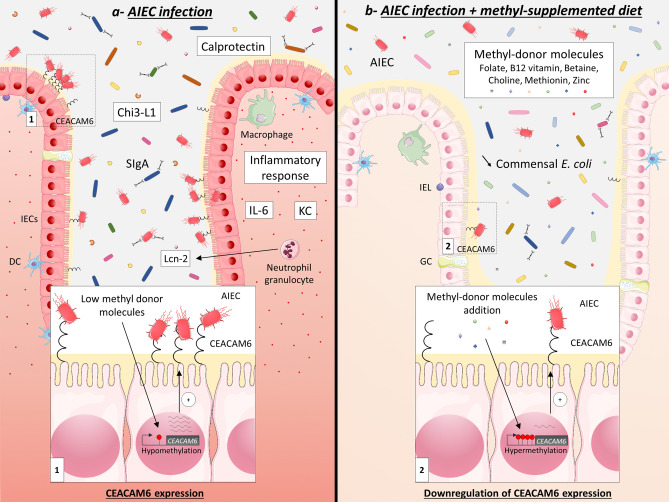
Methyl-donor supplementation in the diet prevents intestinal colonization by AIEC pathobiont. (**a**) In CEABAC10 mice fed a conventional diet, *CEACAM6* promoter is hypomethylated in IECs, which leads to a high expression of *CEACAM6* (insert 1) and, as a consequence, to a great ability of AIEC bacteria to colonize the intestinal mucosa and to induce a pro-inflammatory response (IL-6, KC, Chi3-L1, Calprotectin and Lcn-2). Numerous bacteria are also coated by SIgA in this specific context. (**b**) In CEABAC10 mice fed a methyl-donor-supplemented diet, *CEACAM6* gene promoter is hypermethylated in IECs, which leads to a decrease in its expression level (insert 2) and, as a consequence, to a lower intestinal colonization of the mucosa by AIEC bacteria and to a controlled inflammatory response. Of note, the load of commensal Enterobacteria is profoundly affected by the diet, which is associated to a lower IgA secretion in the lumen. *Chi3-L1* Chitinase 3-Like 1, *DC* dendritic cells, *GC* goblet cells, *IECs* intestinal epithelial cells, *IEL* intraepithelial lymphocytes, *IL-6* interleukin 6, *KC* keratinocyte chemoattractant, *Lcn-2* Lipocalin-2, *SIgA* Secretory Immunoglobulin A. This figure was created using Servier Medical Art templates, which are licensed under a Creative Commons Attribution 3.0 Unported License; https://smart.servier.com.

To conclude, we have demonstrated that methyl-donor molecules enrichment in the diet modulates the expression of genes within intestinal mucosa, with induction of anti-microbial and anti-inflammatory genes and decrease in *CEACAM6* and genes involved in glycosylation pathway. MS diet feeding limits intestinal colonization by pathobiont, such as AIEC bacteria, by setting up an intestinal micro-environment unfavorable to their implantation (Fig. 6). Moreover, we demonstrated an inverse correlation between the endoscopic activity of the disease and the amount of serum folate in patients. These data suggest that it seems important to normalize folate level in CD patients colonized by pathobiont AIEC bacteria. However, further investigations are necessary to identify the better combination of molecules and the doses of methyl-donor molecules to treat Enterobacteria-colonized CD patients and to address possible long-term effects of methyl-donor molecules enrichment.

## Methods

### Crohn’s disease patients

Overall, 28 CD patients were prospectively and consecutively enrolled between September 2015 and September 2016. Clinical parameters including disease location and Crohn’s disease endoscopic index of severity (CDEIS) are detailed in Table 1. Blood samples were taken prior to the endoscopy and sera were used to measure folate (B9 vitamin) concentration with an Advia Centaur folate kit (Chemiluminescence and competition test) using XPT-Centaur analyzer (Siemens, Tarrytown, USA). Samples presenting a maximal rate of 40 ng/ml of folate were included in the study. The samples above this value were considered as outliers and were removed from the study, based on the data from the literature^17,19^. Stools were collected in the morning the day before the endoscopy to reduce intra-individual variations, and were used to measure Calprotectin and Chi3-L1 by chemiluminescence DiaSorin LIAISON Calprotectin assay and Human Chitinase 3-Like 1 ELISA kit (R&D systems; Minneapolis, MN, USA) respectively, according to the manufacturer’s instructions.

### Mice generation, diets and infection

All mice were housed in the animal care facility at the University Clermont Auvergne (Clermont-Ferrand, France). FVB/N WT mice were purchased from Charles River Laboratories and CEABAC10 transgenic mice (heterozygote^30^) were maintained in our animal facilities. WT and CEABAC10 mice were mated to obtain 50% WT mice and 50% CEABAC10 mice. Littermates were used for experimentation.

Two weeks before pregnancy, WT females were fed either standard food (Control diet), or a Methyl-Supplemented diet (MS diet) with enrichment in B12 vitamin, folate, methionine, betaine, zinc sulfate and choline (U8978 version 22, Special Diet Service, Saint Gratien, France) (Supplementary Table S4). The females were bred with transgenic CEABAC10 males. The pups were fed the same diet as their mother until sacrifice (6–8 weeks of age). Four weeks after birth, the offspring were sexed and genotyped.

For infection, mice were pretreated by oral administration of the broad-spectrum antibiotic streptomycin (10 mg intragastric per mouse) to disrupt normal resident bacterial flora in the intestinal tract and were orally challenged 24 h later with 5.10^9^ AIEC LF82 bacteria cultured overnight in LB medium. AIEC LF82 in stools were counted at days 1, 2 and 3 post-infection by homogenization in PBS and numeration on agar plate containing Ampicillin (50 µg/ml) and Erythromycin (20 µg/ml). Three days after infection, mice were sacrificed and ileums and colons were extracted and washed in PBS. One cm was homogenized in 1 ml of physiologic water and serial dilutions were plated on agar plates to count mucosa-associated bacteria and 1 cm was placed in DMEM medium with antibiotics (Gentamicin 50 µg/ml and antibiotic cocktail, PAA) for cytokine release measurement.

### RNA-extraction and RT-qPCR

Total RNA from CEABAC10 colonic mucosa was extracted using Trizol reagent following the manufacturer’s instructions. Briefly, 1 cm of colonic mucosa was homogenized in liquid nitrogen using mortar and pestle. The resulting powder was suspended in 1 ml Trizol reagent (Life Technologies) and 200 µL of chloroform were added. The tubes were vortexed and spin at 12,000 g for 10 min at 4 °C. The aqueous phase was transferred into a new tube and 500 µL of isopropanol were added for 30 min at RT for RNA precipitation. The tubes were spun at 12,000 g for 10 min at 4 °C and the pellet containing RNA was washed twice with 70% ethanol. The pellet was suspended in 50 µL RNase-free water. The RNA quality was assessed by bioanalyzer and their concentration was determined by fluorimeter Qubit 2.0 (Thermo Fisher Scientific) for RT-qPCR or mRNA-sequencing. mRNA were reverse transcribed using PrimeScript RT Reagent kit (Takara) following the manufacturer’s instructions. After cDNA dilution (1/10), 1 µL of cDNA was used as a template for qPCR quantification (iTaq Universal SYBR Green Supermix, Bio-Rad) and 2^−ΔCt^ was applied to determine *CEACAM6* relative expression compared to the housekeeping gene *GAPDH*. Specific primer sequences used are listed in Supplementary Table S5 and each primer pair was designed on two different exons to span a large intronic region.

### mRNA-sequencing and bioinformatic analysis

Libraries were generated from 500 ng of total RNA (RIN 7.40–9.40) as input according to TruSeq Stranded mRNA Sample Preparation, after Poly(A) mRNA purification. Sequencing was performed using NextSeq500, 75 bp Single-read sequencing. Sequenced reads were aligned against the reference genome GRCm38, release 84, using bowtie software (https://bowtie-bio.sourceforge.net) and TopHat (https://ccb.jhu.edu/software/tophat). The 4 human *CEACAMs* genes (*CEACAM3, CEACAM5, CEACAM6* and *CEACAM7*) were added to the reference genome to assess the expression of human *CEACAMs* in the colon. The quality of the sequences was evaluated using Sequencing Analysis Viewer 1.8.37 (Illumina). 85% of reads were over the quality threshold (between 42,734,837 and 77,593,935 good quality reads were obtained). Uniquely mapped RNA-seq data were analyzed with SeqMonk version 1.44.0. Read counts were quantified over exons of merged transcripts using the SeqMonk RNA-seq quantitation pipeline. Differentially expressed genes were identified based on the raw read count quantitation over merged transcript isoforms with the multiple testing corrected DeSeq2 algorithms in SeqMonk. KEGG pathway analysis of misregulated genes was performed using WebGestalt (WEB-based GEne Set AnaLysis Toll kiT; Zhang, B) online software (https://www.webgestalt.org/).

### Western blot

One cm of colonic mucosa was homogenized in liquid nitrogen using mortar and pestle. The powder was suspended in cell lysis buffer [60 mM Tris HCl pH 6.8; SDS 10% v/v, protease inhibitor (miniComplete, Roche)]. The suspension was sonicated (3 cycles of 15 s). Proteins concentration was determined using DC Protein Assay (Bio-Rad). All the samples were adjusted to an equivalent concentration in lysis buffer and the same volume as the sample of denaturation buffer was added [60 mM Tris–HCl pH 6.8, SDS 10% v/v, DTT 0.6% (w/v), Glycerol 20% and bromophenol blue]. The proteins were boiled 10 min at 95 °C before loading 6 µg in a 12% SDS-PAGE gel for electrophoresis migration and transfer to a nitrocellulose membrane (Amersham). After 1 h incubation in blocking buffer (PBS-Tween 20 0.05%, 5% BSA), membranes were blotted with the primary antibodies anti-CEACAM6 (1/2,000) (9A6 clone, Genovac) and anti-GAPDH (1/5,000, Cell Signaling) overnight at 4 °C. Membranes were washed with PBS-T and then incubated with appropriate HRP-conjugated secondary antibodies for 1 h at room temperature. Proteins were detected using ECL (Thermo).

### *E. coli* immunofluorescence

This protocol was adapted from^62^. Snap frozen colon were embedded into optimal cutting temperature (OCT) medium and stored at − 80 °C. Eight micrometers of frozen colon were cut in a cryostat. Colonic sections were fixed in 1% PFA for 20 min, washed in PBS and permeabilization was performed using 0.5X Triton X-100 in PBS for 20 min. Unspecific sites were blocked using PBS with 5% FBS and 2% BSA for 1 h. Goat anti-*E. coli* antibody (Serotec) was diluted in blocking buffer (1/150) and incubated overnight at 4 °C. After 3 PBS washes, tissues were incubated for 90 min with a donkey anti-goat-A488-conjugated secondary antibody diluted in PBS-FBS 5% supplemented with Hoechst. Slides were mounted using Mountex-mounting medium (CellPath). Tissues were visualized using a confocal microscope Zeiss LSM 510 Meta (Carl Zeiss, Inc).

### Cytokine quantification

For cytokine release, 1 cm of colonic mucosa was placed in 1 ml of DMEM medium containing 20 µg/ml gentamicin, 200 U penicillin, 50 mg streptomycin per liter, 0.25 mg amphotericin B per liter, for 24 h and was maintained in an atmosphere containing 5% CO_2_ at 37 °C. The tissues were weighted for standardization. The medium was centrifuged and the supernatant was used for cytokines quantification by ELISA. Lipocalin-2 was quantified in weighted stools as previously described^63^. ELISA experiments were performed using 50 µl of medium-containing released cytokines with kits from R&D systems (KC/CXCL1:DY453, IL-6: DY406, Lcn-2: DY1857) following manufacturer’s instructions.

### By cultural method

Gram negative bacteria were quantified in stools of mice using Drigaslky gelosis. Briefly, stools were weighted and homogenized in 1 ml of PBS for 10 min. The suspension was serially diluted in PBS and 25 µl of each dilution were plated onto Drigalsky gelosis. Colonies were numbered 24 h later and the data were expressed as CFU/g of stool.

### By molecular quantification

Genomic DNA was extracted from mice stools using Nucleospin Soil kit (MACHEREY–NAGEL) following the manufacturer’s instructions. The 16S rDNA was targeted by qPCR for quantification of *E. coli* by TaqMan approach using Dream Taq Master Mix (Thermo Fischer Scientific). The data are expressed as the quantity of 16S *E. coli* relative to total 16S *Eubacteria*. Primers used and PCR mix are specified in Supplementary Table S5.

### Flow cytometry for SIgA-coated bacteria quantification

SIgA were quantified in weighted feces supernatant using a commercially available kit. SIgA-coated bacteria were quantified in the stools of mice as previously described^53^ with minor changes. Briefly, frozen fecal pellets were suspended in ice cold PBS at a concentration of 50 mg/ml and incubated 1 h on ice before incubation for 15 min in disruptor. Samples were spun for 15 min at a speed of 500 g (4 °C). The supernatant, containing bacteria, was collected in a fresh tube and spun for 5 min at a speed of 8,000 g to pellet bacteria. The pellet was suspended in 1 ml staining buffer (1% BSA diluted in PBS) and spun as previously. Pellets were suspended in 100 µL blocking buffer (composed of staining buffer containing 20% FBS), and were incubated on ice for 20 min. One hundred µL of PE-conjugated mouse anti-IgA (IgA Monoclonal Antibody (11-44-2), PE, eBioscience; diluted 1/12.5 in staining buffer) were added to each tube and staining was performed on ice for 30 min. Three washes were performed with staining buffer before analysis on a BD LSRII. Data were then analyzed with BD Facs Diva and FlowJo. The PE positive gate was placed according to isotype control staining.

### Intestinal epithelial cells enrichment

*CEACAM6* specific CpG methylation level was measured on enriched-IECs from colonic mucosa. Colons were flushed and washed 3 times with ice-cold PBS. 0.5 cm colonic sections were incubated in PBS-EDTA 2 mM for 1 h at 4 °C with slow agitation. PBS-EDTA was removed and the colonic sections were washed 3 times with PBS before release of colonic crypts by 1 min hand shaking. The suspension was filtered throughout a 70 µm cell strainer and spun down at 300 g for 10 min. The pellets containing crypts were washed 1 time with PBS and were stored at − 80 °C until DNA extraction.

### Global DNA methylation quantification

Genomic DNA was extracted from intestinal epithelial cells using NucleoSpin Tissue (Macherey–Nagel) following manufacturer’s instructions. Methylation level of LINE-1 was quantified using Global DNA Methylation LINE-1 Kit (Active Motif) following manufacturer’s instructions.

### Bisulfite modification and SnapShot analysis

DNA from IECs was extracted using Nucleospin Tissue extract kit (Macherey–Nagel) following the manufacturer’s instructions. One µg of DNA was subjected to bisulfite modification using CpGenome Fast DNA modification Kit (Millipore) in accordance with the manufacturer’s instructions and as previously described^31^. *CEACAM6* promoter region of interest was amplified using primers described in Supplementary Table S5 (*CEACAM6* promoter modified by bisulfite). Thirty cycles of PCR were performed to amplify the CpG5-containing region of *CEACAM6* promoter, as follows: 1 min 95 °C, 1 min 52 °C and 3 min 72 °C (primers used in Supplementary Table S5). Purification of PCR products was performed using AMPure XP (Beckmann-Coulter, USA). Purified PCR products were then analyzed using a primer extension method  (SnapShot). Extension primers annealed to the amplified DNA template immediately adjacent to CpG5 (Supplementary Table S5). Single nucleotide primer extension was performed in a final volume of 10 μl with 6 μl of purified PCR products, 3 μl of SnapShot reaction mix (Applied Biosystems, Evry, France) and 0.17 µM of a specific primer related to CpG5. Thermocycling conditions were: 94 °C for 5 min followed by 35 cycles of 94 °C for 10 s, 54 °C for 5 s and 60 °C for 10 s. The extended primers labelled with different fluorescent dyes were run on an ABI 3,500 capillary electrophoresis instrument and analyzed with GeneMapper software (Applied Biosystems, Evry, France). Peak area ratios were calculated to measure the relative percentage of methylation. This protocol was adapted from^31^.

### Statistical analysis

Values are expressed as the mean ± SEM of ‘n’ number of experiments or median. Statistical analysis were performed using GraphPad Prism 6 for Windows version 6.07 (GraphPad Software, San Diego, CA, USA) software package for PC. Single comparisons were performed by unpaired Mann–Whitney. One way ANOVA was used when specified. A value of *p* < 0.05 was considered as statistically significant. Two-sided Spearman test was used for correlations analysis.

### Ethical considerations

The study was performed in accordance with the Declaration of Helsinki, Good Clinical Practice and applicable regulatory requirements. The study was approved by IRB “Comité de Protection des Personnes (CPP) Sud-Est 6”—France [approval number AU 904]. All the patients involved in the study have signed a written informed consent. All research was performed in accordance with relevant guidelines. Animal protocols were approved by the Committee for Research and Ethical Issues of the C2E2A (“Comité d’éthique pour l’expérimentation animale Auvergne” N°002, APAFIS#6,448-2017092216023907v1). All experiments were performed in accordance with relevant guidelines and regulations.

## Data availability

The datasets generated during the current study are available in the SRA repository, Bio Project PRJNA641769.

## Supplementary information

Supplementary Legends.

Supplementary Figure 1.

Supplementary Tables.

## Abbreviations

AIEC: Adherent-Invasive *Escherichia coli*
BSA: Bovine serum albumin
CD: Crohn’s disease
CDEIS: Crohn’s disease endoscopic index of severity
CEABAC10: Carcinoembryonic antigen bacterial artificial chromosome 10
CEACAM: Carcinoembryonic antigen-related cell adhesion molecule
FBS: Fetal bovine serum
FITC: Isothiocyanate fluorescein
IBD: Inflammatory Bowel Disease
IL-6: Interleukin-6
KC: Keratinocyte Chemoattractant
kDa: KiloDalton
KEGG: Kyoto encyclopedia of genes and genomes
Lcn-2: Lipocalin-2
MS diet: Methyl-supplemented diet
SIgA: Secretory Immunoglobulin A
WT: Wild type
