# Supplementary material for: Methyl-donor supplementation prevents intestinal colonization by Adherent-Invasive E. coli in a mouse model of Crohn’s disease
Source: Sci Rep. 2020 Jul 31;10:12922. doi: 10.1038/s41598-020-69472-3 (PMC7395125; doi:10.1038/s41598-020-69472-3)
Supplement: Supplementary file 1 — Supplementary Legends. [file 41598_2020_69472_MOESM1_ESM.docx]

***Methyl-donor supplementation prevents intestinal colonization by Adherent-Invasive E. coli in a mouse model of Crohn’s disease***

Gimier Elodie^1^, Chervy Mélissa^1^, Agus Allison^1,2^, Sivignon Adeline^1^, Billard Elisabeth^1^, Privat Maud^3,4^, Viala Sandrine^3,4^, Minet-Quinard Régine^5,6^, Buisson Anthony^1,7^, Vazeille Emilie^1,7^, Barnich Nicolas^1^, Denizot Jérémy^1*^

^*^Corresponding author: Mailing address: M2iSH, UMR 1071 Inserm/Université Clermont Auvergne, CBRV, 28 place Henri Dunant, 63001 Clermont-Ferrand, France. Phone: (33)4 73 17 83 81. Fax: (33) 4 73 17 83 71. *E-mail*: [jeremy.denizot@uca.fr](mailto:jeremy.denizot@uca.fr)

**Online resources: Figure S1 and tables S1-S5 (.xls file)**

**Figure S1**: Uncropped western blots, related to figure 1d

**Table S1**: List of genes down-regulated in colonic mucosa from MS diet group compared to CTR group

**Table S2**: List of genes up-regulated in colonic mucosa from MS diet group compared to CTR group

**Table S3**: Results of the KEGG pathway analysis

**Table S4**: Primers used in this study

**Table S5**: Composition of diets used in this study
