# Supplementary figures and images for: Methyl-donor supplementation prevents intestinal colonization by Adherent-Invasive E. coli in a mouse model of Crohn’s disease
Source: Sci Rep. 2020 Jul 31;10:12922. doi: 10.1038/s41598-020-69472-3 (PMC7395125; doi:10.1038/s41598-020-69472-3)

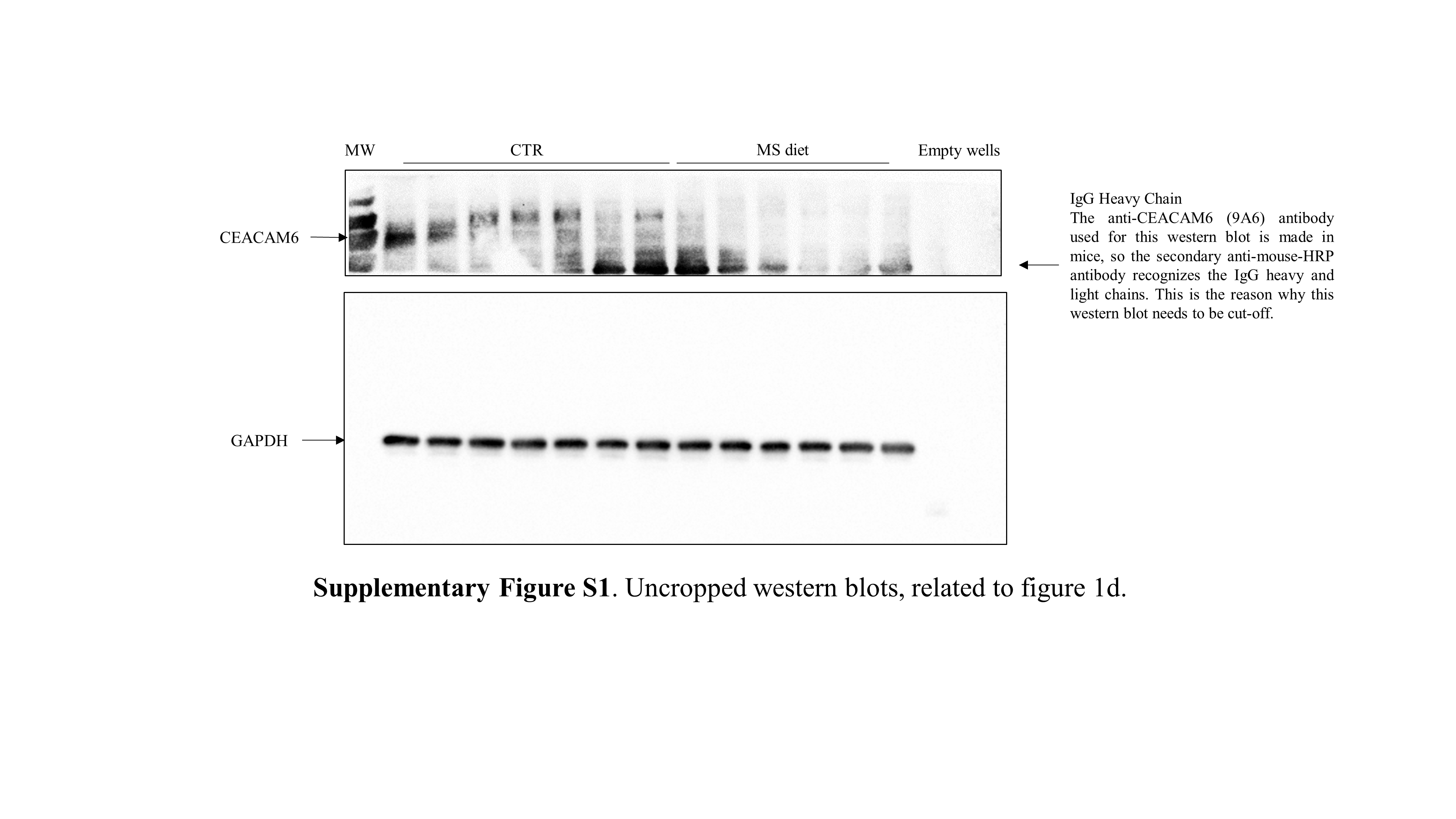

Supplement: Supplementary file 2 — Supplementary Figure 1. [file 41598_2020_69472_MOESM2_ESM.tif]
